# Supplementary material for: Application of the Nutrient-Rich Food Index 9.3 and the Dietary Inflammatory Index for Assessing Maternal Dietary Quality in Japan: A Single-Center Birth Cohort Study
Source: Nutrients. 2021 Aug 19;13(8):2854. doi: 10.3390/nu13082854 (PMC8400739; doi:10.3390/nu13082854)
Supplement: Supplementary file 1 [file nutrients-13-02854-s001.zip › nutrients-1311744-supplementary.pdf]

Figure S1. Sensitivity analysis for breakdown of Nutrient-Rich Food Index 9.3 (NRF9.3) into component scores by excluding the participants with misreported energy intake.

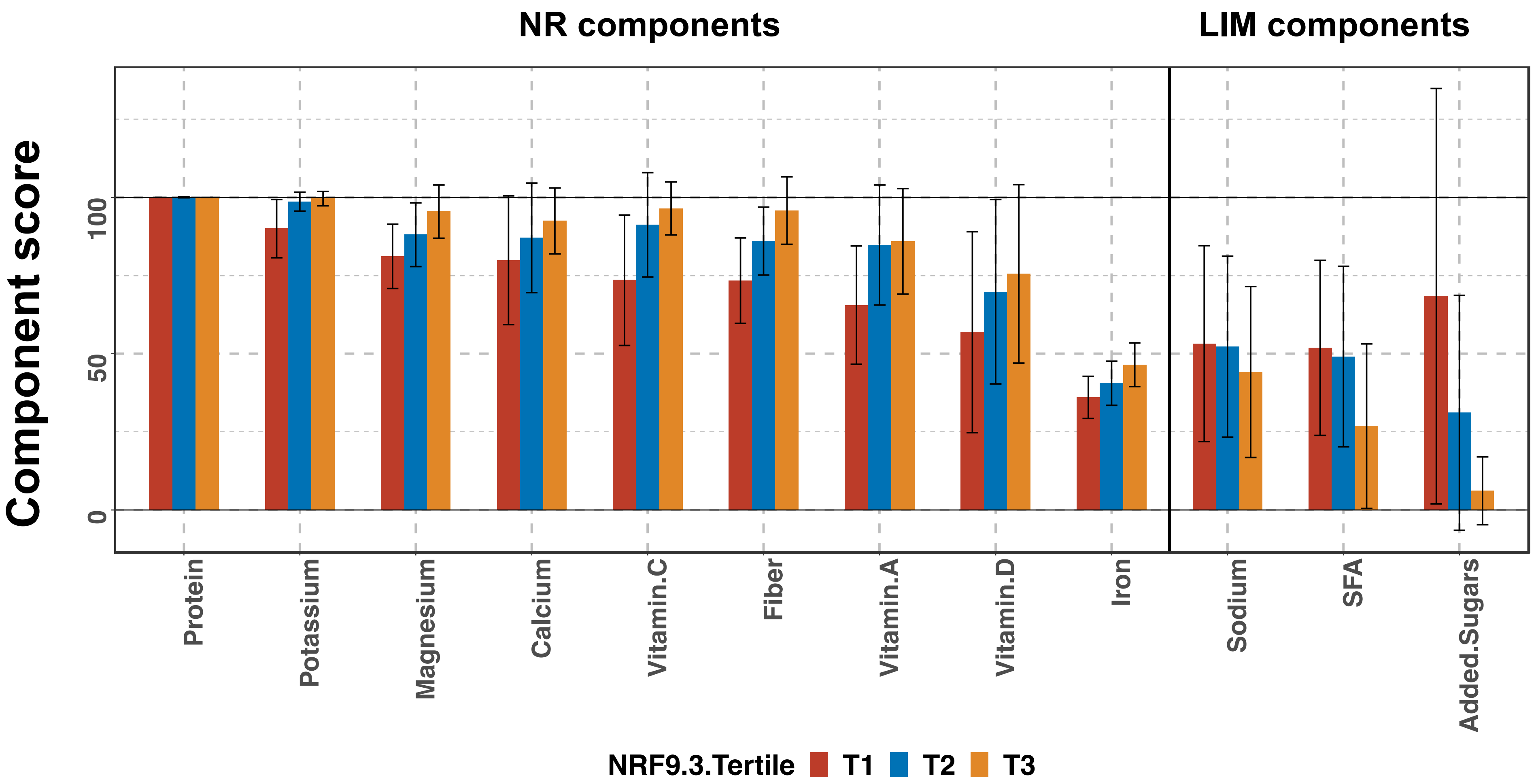

Bars show the mean and error bars represent standard deviation (SD). The nutrients constituting the NR and LIM sub-scores are shown from left to right, from the highest to lowest mean score of the entire cohort. For the NR sub-scores, a higher score indicates a higher dietary quality, while for the LIM sub-scores, a higher score indicates a lower dietary quality. SFA, saturated fatty acids. *n* = 30 (T1), 30 (T2), and 31 (T3).

Figure S2. Sensitivity analysis for breakdown of energy-adjusted dietary inflammatory index (E-DII) into parameter-specific scores by excluding the participants with misreported energy intake.

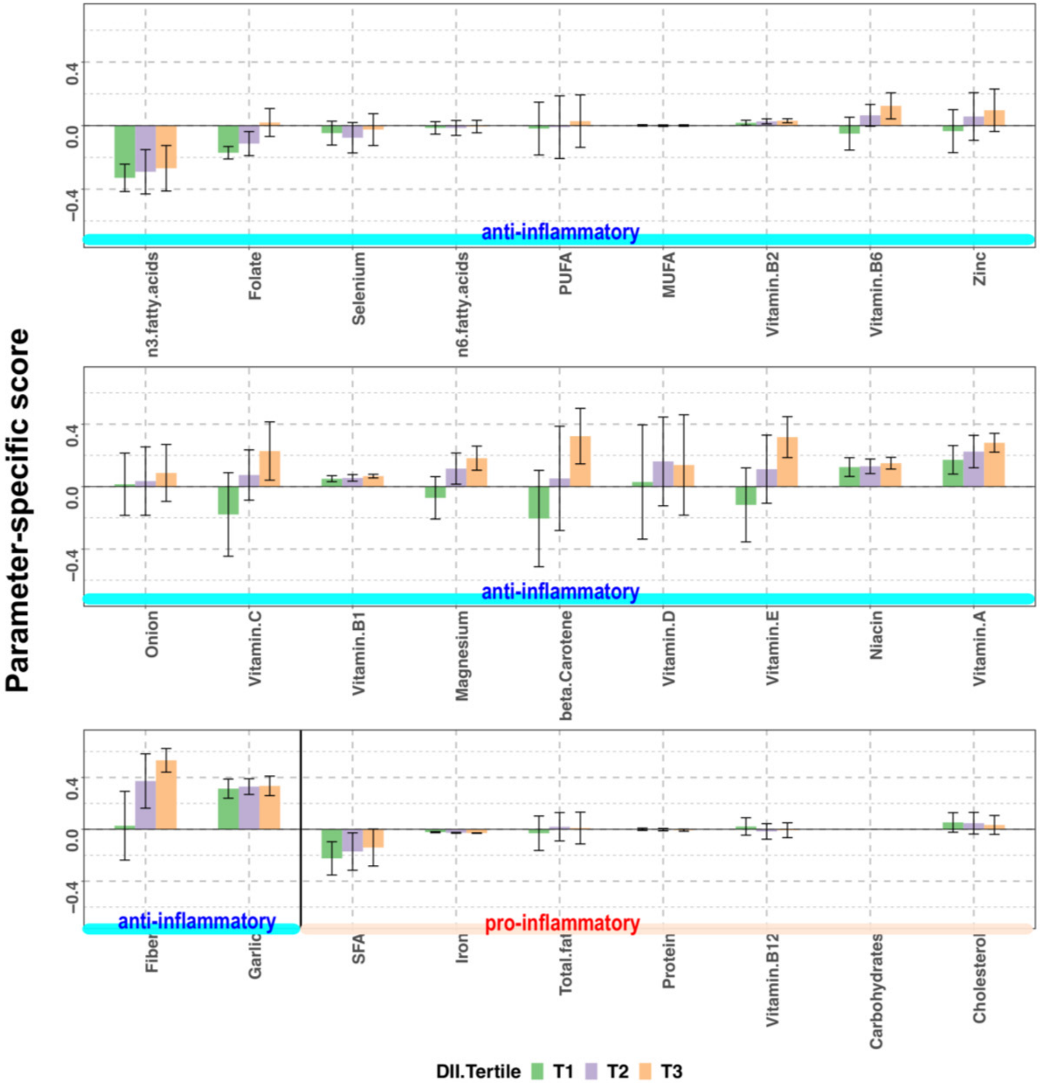

Bars represent the mean and error bars represent standard deviation (SD). The anti-inflammatory and pro-inflammatory parameters constituting the E-DII score are shown from left to right, from the lowest to the highest mean score of the entire cohort. Since the effect score of anti-inflammatory parameters are negative, lower intake of anti-inflammatory nutrients compared to the global mean result in a positive parameter-specific score. In contrast, as the effect score of pro-inflammatory parameters are positive, higher intake of pro-inflammatory nutrients result in a positive parameter-specific score. Higher E-DII scores indicate higher inflammatory potential. SFA, saturated fatty acids; MUFA, monounsaturated fatty acids; PUFA, polyunsaturated fatty acid; n = 30 (T1), 30 (T2), and 31 (T3).

Table S1. Age-specific reference daily values (RDV) for women at mid-gestation for Nutrient-Rich Food Index 9.3 (NRF9.3).

	Age category	18-29 years	30-49 years
Estimated requirement	Energy (kcal/day)[a]	2200	2250
Qualifying nutrients (NR components)	Protein (g)[b]	60	60
	Dietary fiber (g)[c]	18	18
	Vitamin A (ugRAE)[b]	650	700
	Vitamin C (mg)[b]	110	110
	Vitamin D (ug)[d]	7	7
	Calcium (mg)[b]	650	650
	Iron (mg)[b]	21	21.5
	Postassium (mg)[c]	2600	2600
	Magnesium (mg)[b]	310	330
Disqualifying nutrients (LIM components)	Saturated fatty acids (g)[c,e]	17.1	17.5
	Added sugars (g)[f]	27.5	28.1
	Sodium (g NaCl equivalent)[c,g]	7	7

RAE, retinol activity equivalent. [a]Estimated Energy Requirement for moderate level of physical activity for women at mid-gestation. [b]Recommended Dietary Allowance (RDA). [c]Tentative dietary goal for preventing lifestyle-related diseases (DG). [d]Adequate Intake (AI). [e]Determined based on the DG value (7% of energy). [f]Determined based on the World Health Organization's conditional recommendation (5% of energy). [g]7 g NaCl equivalent = 2756 mg sodium.

Table S2. Food parameters of energy-adjusted dietary inflammatory index (E-DII) used in this study.

Parameter used[a]	Global daily mean intake units[a] per 1000 kcal energy intake	Global daily mean intake SD[a] per 1000kcal energy intake	Overall inflammatory effect score[a]	BC-GENIST		
				Mean intake per 1000 kcal energy intake	SD per 1000 kcal energy intake	Mean E-DII value of parameter
Vitamin B12 (ug)	2.50	1.31	0.106	2.74	1.73	-0.001
Vitamin B6 (mg)	0.71	0.36	-0.365	0.66	0.17	0.042
β-Carotene (ug)	1808	837	-0.584	1690	927	0.077
Carbohydrates (g)	132	19	0.097	132	15	-0.001
Cholesterol (mg)	136	25	0.11	177	60	0.048
Total fat (g)	34.73	9.44	0.298	34.53	5.81	-0.005
Dietary fiber (g)	9.14	2.38	-0.663	7.33	1.94	0.306
Folate (ug)	133	34	-0.19	175	58	-0.082
Garlic (g)	2.12	1.41	-0.412	0.23	0.44	0.329
Iron (mg)	6.49	1.80	0.032	3.92	0.84	-0.026
Magnesium (mg)	150.8	67.8	-0.484	138.2	31.1	0.069
MUFAs (g)	13.13	2.97	-0.009	12.80	2.73	0.001
Niacin (mg)	12.60	5.72	-0.246	8.18	2.11	0.131
n-3 Fatty acids (g)	0.52	0.52	-0.436	1.18	0.49	-0.297
n-6 Fatty acids (g)	5.25	3.65	-0.159	5.67	1.32	-0.013
Onion (g)	17.46	8.95	-0.301	16.63	16.09	0.050
Protein (g)	38.62	6.76	0.021	37.44	5.00	-0.003
PUFAs (g)	6.75	1.83	-0.337	6.87	1.55	-0.007
Vitamin B2 (mg)	0.83	0.38	-0.068	0.64	0.14	0.024
Saturated fatty acids (g)	13.91	3.89	0.373	10.81	2.54	-0.185
Selenium (ug)	32.59	12.21	-0.191	38.37	10.87	-0.051
Vitamin B1 (mg)	0.83	0.32	-0.098	0.55	0.13	0.056
Vitamin A (ugRAE)	478.6	252.2	-0.401	261.2	99.5	0.232
Vitamin C (mg)	57.49	21.14	-0.424	56.33	29.01	0.054
Vitamin D (ug)	3.04	1.07	-0.446	2.94	2.44	0.094
Vitamin E (mg)	4.25	0.72	-0.419	3.93	1.13	0.114
Zinc (mg)	4.79	1.07	-0.313	4.58	0.77	0.045

MUFAs, mono-unsaturated fatty acids; PUFAs, poly-unsaturated fatty acids; RAE, retinol activity equivalent; SD, standard deviation. [a]Shivappa N, Steck SE, Hurley TG, Hussey JR, Hébert JR. Designing and developing a literature-derived, population-based dietary inflammatory index. Public Health Nutr. 2014;17(8):1689-96.

Table S3. Overall nutritional characteristics of participants in this study and comparison with the results from the National Health and Nutrition Survey (NHNS) of pregnant women, in Japan.

	Current study (BC-GENIST)			National Health and Nutrition Survey (NHNS), Pregnant women , Japan in 2015			National Health and Nutrition Survey (NHNS), Pregnant women, Japan in 2016			National Health and Nutrition Survey (NHNS), Pregnant women, Japan in 2017		
	mean	SD	median	mean	SD	median	mean	SD	median	mean	SD	median
		n = 108			*n* = 23			*n* = 83			*n* = 31	
Energy (kcal)	1680	277	1673	1713	574	1822	1704	442	1712	1653	311	1644
Protein (g)	62.7	12.4	61.6	65.9	25.2	60.6	58.1	13.3	57.9	60.9	17.2	56.7
Saturated fatty acids (g)	18.24	5.46	18.46	14.36	8.77	12.46	15.79	7.10	15.79	14.20	5.74	13.30
n-6 Fatty acids (g)	9.48	2.51	8.99	9.51	5.02	9.32	9.09	3.35	9.13	10.32	4.16	10.18
n-3 Fatty acids (g)	1.97	0.84	1.83	1.93	1.20	1.64	1.72	0.98	1.48	2.11	1.02	1.86
PFC-F (%)	31.1	5.2	31.1	26.8	7.7	26.1	29.5	6.8	31.2	29.5	5.3	29.3
PFC-C (%)	52.7	6.2	52.7	57.9	8.6	60.0	56.5	7.2	54.8	55.7	6.8	55.3
Dietary fiber (g)	12.3	3.7	11.5	14.2	5.9	12.0	14.0	6.5	13.0	13.3	4.6	11.9
Vitamin A (ugRAE)	442	195	381	521	358	431	506	594	351	445	219	397
Vitamin D (ug)	4.9	4.2	4.1	6.3	7.9	3.4	4.0	5.9	1.9	5.6	7.8	2.8
Vitamin E (mg)	6.6	2.2	6.3	6.7	3.1	6.3	6.4	3.0	6.2	6.2	2.4	5.7
Vitamin K (ug)	232	132	195	205	120	184	233	149	159	297	232	231
Vitamin B1 (mg)	0.93	0.25	0.91	0.79	0.25	0.86	0.80	0.29	0.77	0.77	0.27	0.76
Vitamin B2 (mg)	1.07	0.28	1.01	0.99	0.42	0.97	1.01	0.34	1.01	1.03	0.49	0.87
Niacin (mgNE)	13.7	3.9	13.5	12.2	4.9	11.7	11.1	5.0	9.2	11.4	4.5	11.1
Vitamin B6 (mg)	1.11	0.34	1.09	1.11	0.38	1.19	0.98	0.47	0.87	0.92	0.30	0.86
Vitamin B12 (ug)	4.6	3.0	3.8	3.9	2.9	3.4	3.9	4.2	2.3	4.1	3.1	3.4
Folate (ug)	293	110	281	265	109	263	232	89	220	253	117	236
Pantothenic acid (mg)	5.38	1.31	5.32	5.19	1.79	5.63	5.07	1.68	4.80	4.92	1.77	4.62
Vitamin C (mg)	94	49	82	95	57	91	67	54	54	63	40	48
Potassium (mg)	2231	623	2134	2199	663	2207	1932	792	1782	1864	627	1896
Calcium (mg)	470	171	442	502	269	441	466	188	480	436	242	355
Magnesium (mg)	232	64	221	218	66	232	214	75	197	202	66	189
Phosphorus (mg)	926	208	879	932	338	871	862	230	865	863	285	772
Iron (mg)	6.6	1.7	6.3	6.5	2.4	6.4	6.6	2.5	6.1	6.8	2.3	6.5
Zinc (mg)	7.7	1.9	7.4	7.2	3.5	6.8	7.3	1.9	7.3	7.4	1.8	7.1
Copper (mg)	0.99	0.25	0.97	0.98	0.38	0.95	1.03	0.31	1.02	1.03	0.28	1.03
Sodium (g NaCl equivalent)	7.9	1.8	7.7	9.5	3.1	9.0	8.8	3.3	8.7	8.6	2.6	9.0

RAE, retinol activity equivalent; NE, niacin equivalent; EER, Estimated Energy Requirement; Data of National Institute of Health and Nutrition J. Nutritional Intake Status Survey was obtained from https://www.nibiohn.go.jp/eiken/kenkounippon21/en/eiyouchousa/.

Table S4. Proportion of participants (%) whose intake was less than the Estimated Average Requirement (EAR) or the Adequate Intake (AI) across the tertile (T) categories of Nutrient-Rich Food Index 9.3 (NRF9.3) score.

Nutrients		T1 (n = 36)	T2 (n = 36)	T3 (n = 36)
Energy producing nutrients	Protein (g)[a]	11.1	11.1	0.0
	n-3 Fatty acids (g)[b]	58.3	50.0	30.6
	n-6 Fatty acids (g)[b]	50.0	63.9	36.1
Vitamins	Vitamin A (ugRAE)[a]	86.1	61.1	47.2
	Vitamin D (ug)[b]	83.3	80.6	61.1
	Vitamin E (mg)[b]	80.6	58.3	33.3
	Vitamin B1 (mg)[a]	86.1	75.0	66.7
	Vitamin B2 (mg)[a]	86.1	61.1	58.3
	Niacin (mgNE)[a]	16.7	19.4	5.6
	Vitamin B6 (mg)[a]	86.1	72.2	33.3
	Vitamin B12 (ug)[a]	27.8	22.2	13.9
	Folate (ug)[a]	97.2	88.9	63.9
	Vitamin C (mg)[a]	86.1	52.8	30.6
Minerals	Potassium (mg)[b]	72.2	38.9	8.3
	Calcium (mg)[a]	83.3	69.4	69.4
	Magnesium (mg)[a]	97.2	88.9	44.4
	Iron (mg)[a]	100.0	100.0	100.0
	Zinc (mg)[a]	47.2	41.7	33.3
	Selenium (ug)[a]	0.0	5.6	0.0

[a]Estimated Average Requirement (EAR). [b]Adequate Intake (AI).

Table S5. Proportion of participants (%) whose intake was less than the Estimated Average Requirement (EAR) or the Adequate Intake (AI) across the tertile (T) categories of energy-adjusted dietary inflammatory index (E-DII) score.

Nutrients		T1 (*n* = 36)	T2 (*n* = 36)	T3 (*n* = 36)
Energy producing nutrients	Protein (g)[a]	0.0	11.1	11.1
	n-3 Fatty acids (g)[b]	27.8	44.4	66.7
	n-6 Fatty acids (g)[b]	30.6	58.3	61.1
Vitamins	Vitamin A (ugRAE)[a]	38.9	69.4	86.1
	Vitamin D (ug)[b]	61.1	83.3	80.6
	Vitamin E (mg)[b]	25.0	55.6	91.7
	Vitamin B1 (mg)[a]	58.3	80.6	88.9
	Vitamin B2 (mg)[a]	50.0	69.4	86.1
	Niacin (mgNE)[a]	2.8	16.7	22.2
	Vitamin B6 (mg)[a]	33.3	75.0	83.3
	Vitamin B12 (ug)[a]	11.1	22.2	30.6
	Folate (ug)[a]	55.6	94.4	100.0
	Vitamin C (mg)[a]	30.6	52.8	86.1
Minerals	Potassium (mg)[b]	5.6	38.9	75.0
	Calcium (mg)[a]	61.1	77.8	83.3
	Magnesium (mg)[a]	38.9	91.7	100.0
	Iron (mg)[a]	100.0	100.0	100.0
	Zinc (mg)[a]	33.3	33.3	55.6
	Selenium (ug)[a]	0.0	5.6	0.0

[a]Estimated Average Requirement (EAR). [b]Adequate Intake (AI).
